# A Usefulness of Delta Neutrophil Index (DNI) for Prediction of 28 Day Mortality in Patients with Pneumonia-Induced Sepsis in the Intensive Care Unit

**DOI:** 10.3390/jcm14062002

**Published:** 2025-03-15

**Authors:** SooYoon Moon, YongBum Park, Chang-Won Hong, Sunghoon Park, YunSu Sim, Yousang Ko, SoYoung Park

**Affiliations:** 1 Department of Nephrology, Korea University Guro Hospital, Division of Pulmonary, Allergy and Critical Care Medicine, Seoul 08308, Republic of Korea; sundrop181@hanmail.net; 2 Department of Pulmonary, Allergy and Critical Care Medicine, Kangdong Sacred Heart Hospital, Hallym University College of Medicine, Seoul 05355, Republic of Korea; bfspark2@gmail.com (Y.P.); koyus@naver.com (Y.K.); 3 Department of Physiology, Kyungpook National University, Daegu 41404, Republic of Korea; cwhong@knu.ac.kr; 4 Department of Pulmonary, Allergy and Critical Care Medicine, Department of Internal Medicine, Hallym University Sacred Heart Hospital, Seoul 14068, Republic of Korea; f2000tj@naver.com; 5 Department of Pulmonary, Allergy and Critical Care Medicine, Department of Internal Medicine, Kangnam Sacred Heart Hospital, Hallym University College of Medicine, Seoul 07441, Republic of Korea; sysliver@naver.com

**Keywords:** pneumonia-induced sepsis, septic shock, intensive care unit, twenty-eight-day mortality, delta neutrophil index

## Abstract

**Background**: The delta neutrophil index (DNI) represents the immature granulocyte fraction and is determined by subtracting the fraction of mature polymorphonuclear leucocytes from the sum of myeloperoxidase-reactive cells. The DNI has been proposed as a useful prognostic marker for sepsis. This study evaluated the clinical utility of DNI as a predictive marker in patients with pneumonia-induced sepsis in the intensive care unit (ICU). **Methods**: We conducted a retrospective study of pneumonia-induced sepsis in patients who were admitted to the Kangdong Sacred Heart Hospital’s medical ICUs from July 2022 to March 2024. The DNI was measured on three consecutive days after ICU admission. The primary outcome of this study was a 28-day mortality. **Results**: A total of 227 patients with pneumonia-induced sepsis were included in this study. A 28-day mortality occurred 20.3% of the time in our study. In a univariate analysis, age (*p* = 0.05), lymphocyte (*p* = 0.02), DNI 1 (*p* = 0.01), DNI 2 (*p* = 0.00), DNI 3 (*p* = 0.00), and lactic acid (*p* = 0.00) were significantly associated with 28-day mortality. In a multivariable analysis, lactate (adj. OR: 0.86, 95% CI: 0.78–0.95, *p* = 0.002), and DNI 3 (adj. OR: 0.94, 95% CI: 0.89–0.99, *p* = 0.048) were significantly associated with 28-day mortality. In our study, the most appropriate cut-off values were DNI 1 (7.15), DNI 2 (8.9), and DNI 3 (2.6). Patients with higher DNI 3 (≥2.6) showed higher 28-day mortality than patients with lower DNI 3 values of <2.6 (67.4% vs. 32.6%; *p* < 0.001). However, those aged ≥70 did not show statistically significantly different DNI 1 values between the survivor and non- survivor groups. **Conclusions**: The DNI at 72 h after ICU admission is a promising predictive prognostic marker of 28-day mortality in patients with pneumonia-induced sepsis in the ICU. However, the interpretation of the DNI in sepsis patients aged 70 and older on the first day of hospitalization should be approached with caution.

## 1. Introduction

Mortality and morbidity from pneumonia-induced sepsis remains high despite advances in critical care, understanding of the pathophysiology, and treatment strategies [1,2,3,4,5]. Early recognition and risk stratification are necessary to improve the outcomes in patients with pneumonia-induced sepsis [6,7]. However, definitive and accurate prognostic indicators for this condition have not been found.

Increased percentages of immature granulocytes in systemic circulation are regarded as indicators of increased myeloid cell production and are associated with systemic inflammation [8,9]. However, measurements of this parameter are difficult to obtain in clinical practice because manual measurement is neither accurate nor reproducible [10]. Nahm et al. suggested that the delta neutrophil index (DNI), which represents the differences in leukocyte subfractions assessed by an automated blood cell analyzer, may be useful [11]. The DNI reflects the fraction of circulating immature granulocytes based on the differences between the leukocyte differentials measured in the myeloperoxidase (MPO) reactions and the nuclear lobularity of white blood cells. Recent studies showed a strong correlation between the DNI and the manual immature granulocyte count, in addition to a strong association with disseminated intravascular coagulation scores, positive blood culture rates, and mortality in patients with suspected sepsis [11,12,13,14,15,16,17].

However, little is known about the clinical usefulness of the DNI in assessing the prognosis of patients with pneumonia-induced sepsis in the ICU. In this study, we evaluated the clinical utility of DNI in ICU patients with pneumonia-induced sepsis as an indicator of 28 day-mortality.

## 2. Materials and Methods

### 2.1. Patients

This retrospective study included patients admitted to the medical ICU of Kangdong Sacred Heart Hospital between July 2022 and March 2024. Pneumonia patients with sepsis or septic shock were included. Patients were excluded if they were younger than 18 years or stayed in the ICU for less than 24 h. This research adhered to the principles outlined in the Helsinki Declaration and the protocol was approved by the institutional review board (IRB) of each participating center (IRB no. 2022-03-015-006 from Kangdong Sacred Heart Hospital).

### 2.2. Data Collection

Epidemiological and clinical data available at the time of ICU admission were collected from patients’ medical records. The data included age, sex, comorbid conditions, severity of illness score, laboratory values, and therapeutic interventions performed during their stay in the ICU, such as vasopressor use, renal replacement therapy, or tracheostomy. Furthermore, 28-day mortality and cause of death were evaluated.

Blood samples for the analyses of DNI and other laboratory parameters were obtained within the first 24 h, 48 h, and 72 h after ICU admission. Blood samples were analyzed at the time of ICU admission, and an automatic cell analyzer (ADIVA 2120 Hematology System, Siemens Healthcare Diagnostics, Forchheim, Germany) was used to calculate DNI. This hematologic analyzer is flow cytometry-based and analyzes WBCs by MPO and lobularity/nuclear density channels. After red blood cell lysis, the tungsten-halogen-based optical system of the MPO channel measured cell size and stain intensity in order to count and differentiate granulocytes, lymphocytes, and monocytes based on size and MPO content. Next, the laser diode-based optical system of the lobularity/nuclear density channel countered and classified the cells according to size, lobularity, and nuclear density. The resulting data were inserted in the following formula to determine the DNI:

DNI = leukocyte subfraction assayed in the MPO channel by cytochemical reaction minus the leukocyte subfraction counted in the nuclear lobularity channel by reflected light beam [11].

### 2.3. Definitions

We defined sepsis as “life-threatening organ dysfunction caused by a dysregulated host response to infection”, and septic shock as “a subset of sepsis characterized by particularly profound circulatory, cellular, and metabolic abnormalities, clinically confirmed by the requirement of vasopressors to maintain a mean arterial pressure of 65 mm Hg or greater in the absence of hypovolemia and a serum lactate level greater than 2 mmol/L (>18 mg/dL)” [3,18]. Patient severity was determined using the Acute Physiologic Assessment and Chronic Health Evaluation (APACHE) II Scoring System [19]. We defined DNI 1 as the DNI measured 24 h after ICU admission, DNI 2 as the DNI measured 48 h after ICU admission, and DNI 3 as the DNI measured 72 h after ICU admission.

### 2.4. Statistical Analysis

Continuous variables are expressed as the mean ± standard deviation. Categorical variables were expressed as numbers and percentages. Student’s *t*-test or the Mann–Whitney U test was used to compare continuous variables, whereas the chi-square test or Fisher’s exact test was used to compare categorical variables. Prognostic factors for 28-day mortality were evaluated. Variables with *p* < 0.1 in the univariate analysis were considered in the multivariable analyses to include potential variables with clinical significance. Variables that show significant associations DNI1, DNI2, DNI 3, Lactic acid, lymphocyte—typically based on a predetermined *p*-value threshold—and those deemed clinically relevant are then selected for the multivariable analysis. In the multivariable model, these selected variables are included simultaneously to evaluate their independent effects on the outcome while adjusting for confounding factors. Multivariable logistic regression analysis results were reported as odds ratios and 95% confidence intervals (CIs). Receiver operating characteristic curves (ROC) were constructed, and the area under the curve (AUC) was evaluated. We also evaluated cut-off value of prognostic factor. Our study made an effort to predict the optimum cut point based on time-to-event using the technique of Contal and O’Quigley. All tests were two-sided, and a *p* value of less than 0.05 was considered statistically significant. Data were analyzed using the PASW Statics software version 22 (SPSS Inc., Chicago, IL, USA)

## 3. Results

### 3.1. Patient Characteristics

During the study period, 227 patients who met the inclusion criteria were included in the analysis. The main demographic and clinical characteristics are summarized in Table 1. The mean age was 74 ± 13.7 years. Sixty-six patients were male. The main underlying diseases were diabetes mellitus (124, 54.6%), hypertension (79, 34.8%), and chronic obstructive pulmonary disease (36, 15.9%). A total of 116 (51.1%) patients were diagnosed with septic shock. In total, 129 patients received mechanical ventilation and 25 patients received renal replacement therapy. The median duration of mechanical ventilation was 4.5 (2–80) days. The median duration of ICU stay was 7 (1–90) days. In addition, 46 (51.7%) patients had microbiologically documented pneumonia. Comorbidities were present in 124 SPs (81.5%), with 45 patients having more than two comorbidities. Causative pathogens were identified in 87 SPs (57.2%), with Acinetobacter baumannii being the most common pathogen in patients discharged from hospitals (8.6%), while Streptococcus pneumoniae was the predominant pathogen in community-acquired pneumonia cases (5.2%).

### 3.2. Factors Associated with 28-Day Mortality

The 28-day mortality was 20.3% (46/226). The clinical and laboratory values of survivors and non-survivors are compared in Table 2. In the univariate analysis, age (*p* = 0.05), lymphocyte (*p* = 0.02), DNI 1 (*p* = 0.01), DNI 2 (*p* = 0.00), DNI 3 (*p* = 0.00), combined DNI (*p* = 0.00), and lactic acid (*p* = 0.00) were significantly associated with 28-day mortality (Table 2). However, in multivariate analysis, lactate (adj. OR. 0.86, 95% CI: 0.78–0.95, *p* = 0.002) and DNI 3 (adj. OR. 0.94, 95% CI: 0.89–0.99, *p* = 0.048) were the risk factors for 28-day mortality (Table 3). We analyzed the cut-off level for the DNI value on the first day of treatment for sepsis patients (DNI 1). Using a cut-off value of 7.15%, the accuracy, sensitivity, specificity, positive predictive value (PPV), and negative predictive value (NPV) of the DNI 1 in sepsis were found to be 73%, 80%, 46%, 85%, and 37%, respectively. We also analyzed the cut-off level for the DNI value on the second day of treatment (DNI 2) for sepsis patients. When the cut-off for DNI 2 is set at 8.9%, the accuracy, sensitivity, specificity, positive predictive value (PPV), and negative predictive value (NPV) of the DNI 2 in sepsis were found to be 81%, 88%, 49%, 87%, and 51%, respectively.

**Figure 1:**
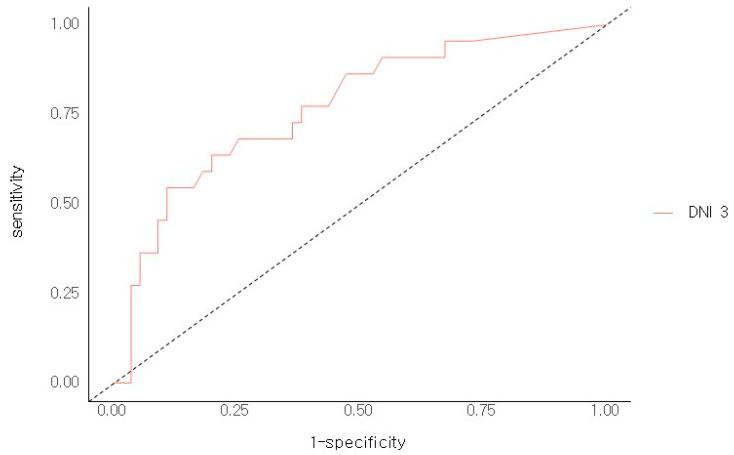
ROC Analysis of DNI 3 for 28 days mortality. When the Threshold > 3, Sensitivity was 0.64, Specificity was 0.8, accuracy was 0.75, positive predictive value 0.56, negative predictive value was 0.85.

**Figure 2:**
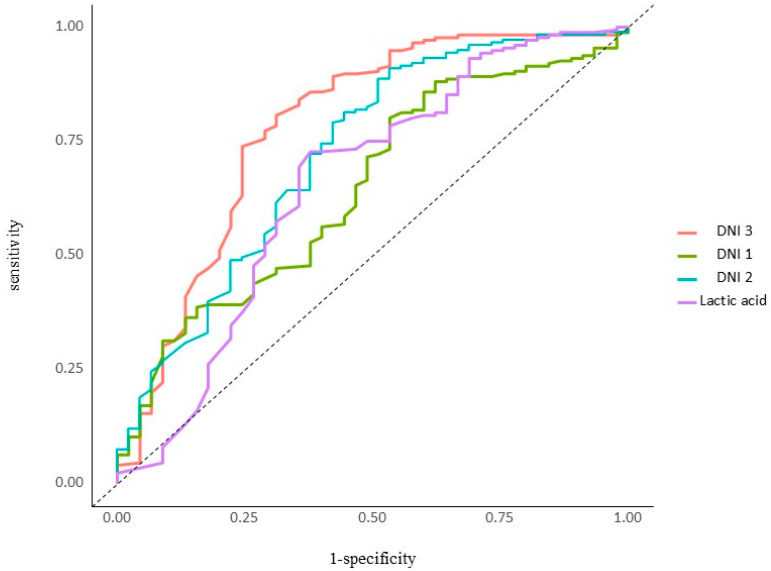
ROC Analysis of DNI 1, DNI 2, DNI 3, Lactic acid for 28 days mortality.

In the higher DNI 3 group (≥2.6), 67.4% of the patients died within 28 days, whereas in the lower DNI 3 group (<2.6), 32.6% of patients died during the first 28 days (*p* = 0.00). Using a cut-off value of 2.6%, the sensitivity, specificity, positive predictive value (PPV), and negative predictive value (NPV) of the DNI 3 in sepsis were found to be 69%, 73.9%, 77.9%, and 64.1%, respectively (Figure 1). DNI 3 was a more specific predicter for 28-day mortality than the other variables. The area under the curve of DNI 3 was 0.781 (95% CI 0.694–0.868), whereas for DNI 1 it was (0.647, 95% CI 0.558–0.736), for DNI 2 it was (0.721, 95% CI 0.63–0.81), and for lactic acid it was (0.655, 95% CI 0.553–0.756) (Figure 2).

### 3.3. Subgroup Analysis of Delta Neutrophil Index (DNI) as Predictor of 28-Day Mortality

The patients were classified into subgroups according to age (≥70 or <70). Interestingly, the ≥70 age group did not show statistically significant difference in DNI 1 values between the survivor and non-survivor groups in the univariate analysis. The <70 age group showed statistically significant differences in DNI 1 values between the survivor and non-survivor groups, while DNI 2 and DNI 3 showed statistically significantly different DNI 1 values between the survivor and non-survivor groups for all ages (Table 4).

## 4. Discussion

This study showed that the DNI value, which reflects the number of circulating granulocyte precursors in the blood, at 72 h after ICU admission, can be a useful prognostic factor for 28-day mortality in patients with pneumonia-induced sepsis. In our study, the DNI was higher in the non-survivor group than in the survivor group throughout the treatment period, although statistical significance was confirmed only at 72 h from ICU admission. We conducted the previous study in the General ICU at Chuncheon Sacred Heart Hospital between January 2012 and February 2013. This study included 89 patients with UTI sepsis, skin and soft tissue infection, and post-operative sepsis. During the study period, 89 patients who met the inclusion criteria were included in the analysis. The main demographic and clinical characteristics are summarized in Supplement 1. The median age was 78 (66.5–84) years. Nearly half of the patients were male (53, 59.6%). The 28-day mortality was 29.2% (26/89) (Supplementary Table S1). In multivariate cox proportional analysis, septic shock (odds ratio (OR) of 0.012; 95% CI of 0.001–0.188; *p* = 0.002) and DNI 3 (OR 1.708; 95% CI 1.010–1.150; *p* = 0.048) were the risk factors for 28-day mortality. In the higher DNI 3 group (≥3), 53.8% (14/26) of the patients died within 28 days, whereas in the lower DNI 3 group (<3), only 15.7% (8/51) died during the first 28 days (*p* = 0.00). When defining the DNI 3 threshold as 3.0, the sensitivity was 0.64, specificity was 0.8, positive predictive value (PPV) was 0.65, negative predictive value (NPV) was 0.85, and the AUC was 0.79 (95% CI 0.651–0.887) (Supplement 2).

A total of 227 patients with pneumonia-induced sepsis were included in this study. The 28-day mortality was 20.3% in our study. In a univariate analysis, age (*p* = 0.05), lymphocyte (*p* = 0.02), DNI 1 (*p* = 0.01), DNI 2 (*p* = 0.00), DNI 3 (*p* = 0.00), and lactic acid (*p* = 0.00) were significantly associated with 28-day mortality. In a multivariable analysis, lactate (adj. OR: 0.86, 95% CI: 0.78–0.95, *p* = 0.002) and DNI 3 (adj. OR: 0.94, 95% CI: 0.89–0.99, *p* = 0.048) were significantly associated with 28-day mortality. In our study, the most appropriate cut-off values were DNI 1 (7.15), DNI 2 (8.9), and DNI 3 (2.6). Patients with higher DNI 3 (≥2.6) showed higher 28-day mortality than patients with lower DNI 3 values of <2.6 (67.4% vs. 32.6%; *p* < 0.001). Since this cohort had a very high mortality rate, the application of the prognostic marker may not be suitable for other ICUs. The DNI 1 and DNI 2 are also much higher than the DNI 3, and even higher than the cut-off value 3. It is often difficult to determine between Day 1 or Day 3 because some patients enter ICU on time whereas some may be delayed. Therefore, we analyzed the cut-off level for the DNI value on the first day of treatment for sepsis patients (DNI 1). Using a cut-off value of 7.15%, the accuracy, sensitivity, specificity, positive predictive value (PPV), and negative predictive value (NPV) of the DNI 1 in sepsis were found to be 73%, 80%, 46%, 85%, and 37%, respectively. We also analyzed the cut-off level for the DNI value on the second day of treatment (DNI 2) for sepsis patients. When the cut-off for DNI 2 is set at 8.9%, the accuracy, sensitivity, specificity, positive predictive value (PPV), and negative predictive value (NPV) of the DNI 2 in sepsis were found to be 81%, 88%, 49%, 87%, and 51%, respectively. These findings agree with some previous reports. An elevated DNI often reflects a heightened immune response due to the body’s attempt to combat severe infection. Research suggests that DNI is a valuable early indicator in sepsis as it may help differentiate sepsis from other non-infectious conditions and provide a quick estimate of the severity of infection. Higher DNI levels have been associated with increased severity of sepsis, poorer patient outcomes, and even higher mortality rates [5,7,20,21,22,23,24,25,26,27].

Recent studies suggest that sepsis impairs the innate immunity of patients [28,29]. Neutrophil paralysis in sepsis results in the failure of neutrophils to migrate to the site of infection and causes inappropriate neutrophil sequestration in remote organs [30]. We proposed that neutrophil paralysis in sepsis may cause a rapid and early production of immature neutrophils to compensate for the deficiency of active neutrophil. Since higher DNI levels correlate with increased numbers of immature neutrophils, patients with higher DNI levels may have more dysregulated immune functions. Those patients who maintain a high DNI until 72 h after the start of treatment may have sustained dysregulation of immunity. Thus, patients with higher DNI levels may not be responding well to treatment for pneumonia-induced sepsis. Therefore, DNI at 72 h could be an alarming marker to check the patient’s status again and to consider other treatment strategies.

However, DNI cut-off for predicting mortality varied. In our study, the optimal DNI cut-off for predicting mortality was 3%. The higher DNI group (≥2.6), measured 72 h after ICU admission, showed significantly higher 28-day mortality (*p* = 0.00) than the lower DNI group (<2.6). Similarly, Lee et al. reported that a DNI cut-off of 2% at 72 h after onset of neonatal sepsis was associated with the 7-day mortality rate [31]. However, a previous study by Kim et al. reported that the optimal DNI cut-off for predicting mortality was 7.6% in patients with Gram-negative bacteremia [20]. Furthermore, Park et al. reported that a DNI > 6.5% was a good predictor of severe sepsis and septic shock within 24 h of admission to an ICU [13]. Therefore, further evaluation of the adequate cut-off value of DNI is needed.

We demonstrated that the DNI correlated with the severity of pneumonia-induced sepsis in the ICU. DNI values were higher in the septic shock group compared to the sepsis group. Previously, Park et al. showed that DNI may be used as a marker of disease severity in critically ill patients with sepsis [13]. Given that the process of granular leukocyte differentiation starts from immature granulocyte formation, the change in DNI may have preceded the change in absolute numbers of WBCs or neutrophils, thus contributing to predicting the development of septic shock. Therefore, it is important for clinicians to identify patients who are at risk of developing septic shock before the signs of organ dysfunction or circulatory failure appear.

Interestingly, in our study, no statistical significance was found in the DNI 1 between the survival group and the non-survival group of elderly patients (≥70). This could be explained by several factors related to the immune system and its response in aging individuals. The first theory is the immune depression in older people. Immunosenescence refers to the gradual deterioration of the immune system associated with aging. In older individuals, both the innate and adaptive immune responses tend to weaken, which means that their bodies may not mount the same level of response to infection as younger people do [32]. A lower DNI in older adults could be due to a decreased production of neutrophils, particularly immature granulocytes, during infections or inflammatory processes. The bone marrow in older individuals may not respond as robustly to signals that normally stimulate neutrophil production [33]. The second theory is that older people often experience chronic low-grade inflammation, sometimes referred to as “inflammaging”. This persistent, low-level inflammation might lead to a baseline activation of the immune system, which can mask or reduce the body’s capacity to produce a surge of neutrophils in response to acute infections [34]. This state of chronic immune activation could potentially lead to lower increases in immature neutrophils, resulting in a lower DNI when acute infection occurs. The third theory is that older adults often have multiple comorbid conditions (e.g., diabetes, chronic kidney disease) that can affect their immune response and ability to produce neutrophils. Certain medications commonly used in older populations, such as immunosuppressants or corticosteroids, can also blunt the body’s inflammatory response and neutrophil production. These factors can contribute to a lower DNI in older patients, as their overall immune response may be suppressed or altered by both their underlying conditions and treatments.

In our study, the median lymphocyte value was 15.4 ± 15.3. In univariate analysis lymphocyte was associated with 28-day mortality (*p* = 0.016, OR (95% CI) 0.98 (0.96–1)). However, in multivariable analysis, lymphocyte was not associated with 28-day mortality. (*p* = 0.29, OR 0.99 (0.96, 1.01). Lymphocytes are pivotal orchestrators of the immune response in sepsis, balancing infection-fighting inflammation with anti-inflammatory regulation [33]. During early sepsis, there is often a surge of pro-inflammatory cytokines (within the first 24–48 h), followed by a compensatory anti-inflammatory shift. This balance, however, becomes dysregulated; a hallmark of sepsis is a breakdown of lymphocyte homeostasis, characterized by excessive lymphocyte activation followed by sepsis-induced lymphopenia [34]. Profound lymphocyte depletion is very common in septic ICU patients and underpins sepsis-related immune dysfunction. The degree and duration of lymphocyte depletion have significant prognostic value in sepsis. Importantly, the persistence of lymphopenia (rather than just its presence on day 0) is a critical determinant of outcomes [35]. Recently, Zhibin Wang suggested that the “immunosenescence phenomenon” induced by sepsis-related lymphopenia might explain the dynamic changes in lymphocyte numbers, describing a series of intrinsic processes from cellular reduction to proliferation impediments [35]. However, our study did not evaluate lymphocyte subtypes, and the sample size was too small, so we believe that further research is necessary.

Our study has the following strengths. Although there has been substantial research on DNI, studies specifically on pneumonia sepsis are limited. This study demonstrated that DNI values could be significant in predicting outcomes for patients with pneumonia sepsis. Notably, DNI was measured sequentially on days 1, 2, and 3, and the third-day value was shown to have predictive significance for prognosis. This suggests that continuous measurement of DNI may be practically necessary. Additionally, this study indicates that DNI interpretation may differ in elderly patients, emphasizing the importance of serial follow-up for older patients.

Although our study suggests the prognostic value of DNI in pneumonia-induced sepsis patients, several limitations exist. Firstly, this study was conducted retrospectively in a single center; therefore, the possibility of selection bias remains. Secondly, the elevation of immature granulocytes is not specific for infection and may be observed in various other conditions such as myeloproliferative disorder, chronic inflammatory disorders, tissue damage, acute hemorrhage, and neoplasia. Thirdly, because we only evaluated short-term mortality, it is still questionable whether DNI 3 can predict long-term outcomes in patients with pneumonia-induced sepsis. Therefore, more studies with a larger number of patients are required to validate the clinical usefulness of DNI as a severity and prediction marker of pneumonia-induced sepsis.

## 5. Conclusions

These data shed new light on the role of the DNI in pneumonia-induced sepsis. DNI measured 72 h after ICU admission may serve as a useful prognostic marker for 28-day mortality in patients with pneumonia-induced sepsis in the ICU. Especially, patients with higher DNI 3 (≥2.6) showed higher 28-day mortality than patients with lower DNI 3 (<2.6) group. In our study, the most appropriate cut-off values were DNI 1 (7.15), DNI 2 (8.9), and DNI 3 (2.6). Early detection and treatment initiation is essential for improving the treatment outcome in pneumonia-induced sepsis. Therefore, the identification of reliable biomarkers for diagnosis and guidance of treatment in sepsis patients is required. Our data show the usefulness of DNI at 72 h as a prognostic marker in patients with pneumonia-induced sepsis in the ICU. Based on these results, we suggest that increased DNI value should alert clinicians to apply more aggressive therapy.

## Figures and Tables

**Table 1:** Baseline characteristics of the patients with pneumonia sepsis.

Variables	No. of Patients (%) or Mean ± SD (n = 227)
Age	74 ± 13.7
Sex (Male)	150 (66.1%)
Septic shock	116 (51.1%)
Cormobidities	
DM	124 (54.6%)
HTN	79 (34.8%)
Heart Disease	32 (14.1%)
Stroke	14 (6.2%)
COPD	36 (15.9%)
IPF *	12 (4.8%)
Dementia	24(10.6%)
Chronic Liver Disease	27 (11.9%)
Solid cancer	32 (14.1%)
Severity at ICU admission	
APACHE II	22.0 ± 5.9
Treatment in ICU	
Mechanical ventilation	129 (56.8%)
Tracheostomy	87 (38.3%)
CRRT	25 (11.0%)
ECMO	5 (2.2%)
ILA	4 (1.7%)
**Laboratory findings**	**No. of Patients (%) or Mean ± SD**
WBC (10^3^/uL)	13,227.8 ± 7864.3
Hb (g/dL)	11.8 ± 3.1
Platelet (10^3^/uL)	244.6 ± 133.9
Neutrophil (%)	79.1 ± 16.5
DNI 1 (%)	6.9 ± 12.9
DNI 2 (%)	5.9 ± 12.0
DNI 3 (%) Combined DNI (%)	4.9 ± 12.5 17.6 ± 33.5
Lymphocyte (%)	15.4 ± 15.3
Na (mEq/L)	137.2 ± 10.0
K (mEq/L)	4.2 ± 0.8
BUN (mg/dL)	28.4 ± 21.0
Cr (mg/dL)	1.4 ± 2.2
AST (IU/L)	67.4 ± 213.9
ALT (IU/L)	41.5 ± 123.1
BNP (pg/mL)	371.7 ± 564.5
CRP (mg/dL)	125.4 ± 108.6
Procalcitonin (ng/mL)	5.2 ± 16.9
Lactic acid (mmol/L)	3.4 ± 3.8

Definition of abbreviations: APACHE: Acute Physiology and Chronic Health Evaluation MV: Mechanical Ventilation, CRRT: Continuous renal replacement therapy ECMO: Extracorporeal membranous oxygenation, ILA: Interventional lung assist, WBC: White blood cell, DNI 1: Delta neutrophil index at 24 h of ICU admission, DNI 2: Delta neutrophil index at 48 h of ICU admission, DNI 3: Delta neutrophil index at 72 h of ICU admission, Hb: Hemoglobin, Na: Sodium, K: Potassium, BUN: Blood urea nitrogen, Cr: Creatinine, AST: Aspartate aminotransferase, ALT: Alanine aminotransferase, BNP: Brain natriuretic peptide CRP: C- reactive protein. Demographics and clinical outcomes are presented for the 227 selected samples. The results are expressed as mean SD for continuous variables and as number (%) for categorical variables. All values were obtained at the time of admission to the intensive care unit. * Solid cancer included lung cancer, breast cancer, colon cancer, stomach cancer, pancreatic cancer and ovary cancer.

**Table 2:** Univariable analysis in Survivor vs. Nonsurvivor in 28 days mortality.

Variables	Survivors (N = 181)	Non Survivors (N = 46)	*p*-Value
Age	73.1 ± 13.8	77.6 ± 13.1	0.05
Sex (Male, %)	115 (63.5)	35 (76.1)	0.15
Septic shock	98 (54.1)	28 (60.9)	0.09
Severity scores			
APACHE	21.7 ± 5.9	23.3 ± 6.1	0.10
Treatment in ICU			
MV	97 (53.6)	32 (69.6)	0.07
CRRT	19 (10.5)	22 (47.8)	0.82
ECMO	2 (1.1)	2 (4.3)	0.57
ILA	3 (1.6)	2 (4.3)	0.64
Tracheostomy	69 (38.1)	18 (39.1)	0.98
Laboratory findings			
WBC (10^3^/uL)	12,998.0 ± 7834.8	14,132.0 ± 8001.2	0.39
Neutrophil (%)	80.5 ± 14.5	73.6 ± 22.0	0.05
DNI 1 (%)	5.8 ± 12.4	11.4 ± 13.8	0.01
DNI 2 (%)	3.8 ± 9.2	14.5 ± 17.1	0.00
DNI 3 (%)	2.3 ± 8.6	14.9 ± 19.0	0.00
Combined DNI (%)	4.6 (0.6–12.3)	21.5 (5.7–67.0)	0.00
Lymphocyte	13.1 ± 8.6	10.0 ± 4.8	0.04
Hb (g/dL)	11.8 ± 3.2	11.5 ± 2.5	0.48
Platelet(10^3^/uL)	248.3 ± 138.0	229.9 ± 116.8	0.36
Na (mEq/L)	136.9 ± 9.9	138.4 ± 10.4	0.39
K (mEq/L)	4.6 ± 0.9	4.1 ± 0.8	0.06
BUN (mg/dL)	36.8 ± 25.0	26.3 ± 19.3	0.07
Cr (mg/dL)	1.5 ± 1.3	1.4 ± 2.4	0.53
AST (IU/L)	140.5 ± 460.2	48.8 ± 52.8	0.18
ALT (IU/L)	68.0 ± 226.5	34.8 ± 77.3	0.33
BNP (pg/mL)	128.5 ± 106.5	124.7 ± 109.4	0.83
CRP (mg/dL)	6.0 ± 14.6	5.0 ± 17.5	0.69
Procalcitonin (ng/mL)	446.8 ± 546.4	352.3 ± 569.0	0.32
Lactic acid (mmol/L)	5.5 ± 5.6	2.8 ± 2.9	0.00

Definition of abbreviations: APACHE: Acute Physiology and Chronic Health Evaluation MV: Mechanical Ventilation, NIV: Non invasive Ventilation CRRT: Continuous renal replacement therapy ECMO: Extracorporeal membranous oxygenation, ILA: Interventional lung assist, BP: Blood pressure HR: Heart rate RR: Respiratory rate BT: Body temperature, WBC: White blood cell, DNI 1: Delta neutrophil index at 24 h of ICU admission, DNI 2: Delta neutrophil index at 48 h of ICU admission, DNI 3: Delta neutrophil index at 72 h of ICU admission, Hb: Hemoglobin, Na:Sodium, K: Potassium, BUN:Blood urea nitrogen, Cr: Creatinine, AST:Aspartate aminotransferase, ALT:Alanine aminotransferase, BNP: Brain natriuretic peptide CRP: C- reactive protein. Demographics and clinical outcomes are presented for the 227 selected samples. The results are expressed as mean SD for continuous variables and as number (%) for categorical variables. All values were obtained at the time of admission to the intensive care unit. Solid cancer included lung cancer, breast cancer, colon cancer, stomach cancer, pancreatic cancer and ovary cancer

**Table 3:** Multivariable analysis of predictive factors for 28-day mortality.

Variables	Odds Ratio (95% CI)	*p*-Value
Age	0.97 (0.94, 1.0)	0.03
Sex	1.98 (0.81, 4.81)	0.13
Lymphocyte	0.99 (0.96,1.01)	0.29
DNI 1	1.04 (0.98, 1.1)	0.17
DNI 2	0.97 (0.9, 1.05)	0.51
DNI 3	0.95 (0.89, 0.99)	0.05
Lactic acid	0.86 (0.78, 0.95)	0.00

DNI 1: Delta neutrophil index at 24 h of ICU admission, DNI 2: Delta neutrophil index at 48 h of ICU admission, DNI 3: Delta neutrophil index at 72 h of ICU admission,.

**Table 4:** Age -subgroup analysis of DNI for 28 days mortality.

	Coeff. (95%CI)	*p* Value
DNI 1		
≥70	−4.23 (−9.2, 0.73)	0.097
<70	−11.67 (−19.6, −3.74)	0.005
DNI 2		
≥70	−8.93 (−13.43, −4.43)	<0.001
<70	−19.38 (−25.83, −12.94)	<0.001
DNI 3		
≥70	−10.47 (−14.74, −6.2)	<0.001
<70	−23.76 (−31.67, −15.86)	<0.001

DNI 1: Delta neutrophil index at 24 h of ICU admission, DNI 2: Delta neutrophil index at 48 h of ICU admission, DNI 3: Delta neutrophil index at 72 h of ICU admission,.

## Data Availability

The datasets are available from the corresponding author on reasonable request.

## Supplementary Materials

The following supporting information can be downloaded at: https://www.mdpi.com/article/10.3390/jcm14062002/s1, Table S1. Baseline characteristics of the patients with pneumonia sepsis. Table S2. Univariable analysis in Survivor vs. Nonsurvivor in 28 days mortality. Table S3. Cox proportional hazard analysis of predictive factor for 28-day mortality. Figure S1. ROC analysis of DNI 3 for 28-day mortality.
