# Supplementary material for: A Usefulness of Delta Neutrophil Index (DNI) for Prediction of 28 Day Mortality in Patients with Pneumonia-Induced Sepsis in the Intensive Care Unit
Source: J Clin Med. 2025 Mar 15;14(6):2002. doi: 10.3390/jcm14062002 (PMC11942979; doi:10.3390/jcm14062002)
Supplement: Supplementary file 1 [file jcm-14-02002-s001.zip › supplement 1.pdf]

Table S1. Baseline characteristics of the patients with pneumonia sepsis

Variables	Values (n=89)
Age	78 (66.5-84)
Sex (Male)	53(59.6%)
Comorbidity	
DM	49(55.1%)
HTN	31(34.8%)
TB	12(13.5%)
Heart Disease	13(14.6%)
Stroke	5(5.6%)
COPD	14(15.7%)
IPF*	3(3.4%)
Dementia	8(8.9%)
Chronic alcoholics	11(12.4%)
Solid cancer	6(6.7%)
Severity on ICU admission	
SOFA	8(7-11.5)
APACHE II	24 (19.5-29.0)
Treatment in ICU	
Vasopressors or Inotropics	41(46.1%)
MV	62(69.7%)
Tracheostomy	14(15.7%)
CRRT	13(14.6%)
ECMO	4 (4.5%)
ILA	4(4.5%)

Data are presented as the median(interqurtile range(IQR)) for continuous variables and as the number(%) for the categorical variables.
SOFA: Sequential Organ Failure Assessment, APACHE: Acute Physiology and Chronic Health Evaluation
MV: Mechanical Ventilation, CRRT: Continuous renal replacement therapy ECMO: Extracorporeal membranous oxygenation, ILA: Interventional lung assist, BP: Blood pressure HR: Heart rate RR: Respiratory rate BT: Body temperature Hb: Hemoglobin, Hct: Hematocrit Cr: Creatinine, BNP: Brain natriuretic peptide CRP: C- reactive protein

Table S2. Univariable analysis in Survivor vs Nonsurvivor in 28 days mortality

Variables	Survivors (n=63)	Non survivors (n=26)	p-value
Age	80(66-84)	75(66.7-84.2)	0.65
Sex (Male, %)	35(55.6%)	18(69.2%)	0.24
Septic shock	21(33.3%)	20(76.9%)	0.001
Severity scores			
SOFA	8.0(7.0-9.0)	10.5(8.0-13.0)	0.001
APACHE	22.0(19.0-26.0)	28.0(21.5-34.0)	0.07
Treatment in ICU			
Inotropics	22(34.9%)	18(69.2%)	0.001
MV	38(60.3%)	24(92.3%)	0.002
CRRT	4(3.3%)	9(34.6%)	0.001
Tracheostomy	11(17.5%)	3(11.5%)	0. 54
Vital Sign			
Mean BP	80.0(63.0-90.0)	71.0(50-82.5)	0.13
HR	107.5(93.7-123.2)	113.0(90.0-143.5)	0.19
RR	25.0(20.0-29.2)	27.0(24.0-34.0)	0.10
BT	36.9(36.5-37.4)	36.8(35.3-37.2)	0.06
Laboratory findings			
WBC(10^3/ul)	10.7(6.8-15.3)	10.3(6.1-19.3)	0.93
Neutrophil (%)	85.2(76.7-90.5)	83.7(77.6-90.1)	0.92
DNI 1(%)	2.75(1.4-5.5)	4.1(2.0-23.1)	0.21
DNI 2(%)	2.6(1.2-9.1)	7.3(1.4-21.3)	0.15
DNI 3 (%)	1.2(0.0-2.7)	6.0(1.6-12.1)	0.005

Hb (g/dl)	12.1(11.1-13.6)	11.7(9.3-14.2)	0.45
Platetlet(10^3/ul)	241.0 (166.2-301.1)	166.5(88.5-303.2)	0.07
Na (mEq/L)	137.0(132.0-139.0)	136.5(131.7-142.0)	0.54
K(mEq/L)	4.2(3.8-4.6)	4.4(3.9-4.9)	0.19
BUN (mg/dl)	23.8(12.8-35.9)	26.3(19.5-47.7)	0.29
Cr(mg/dl)	1.2(0.9-1.6)	1.2(0.6-1.9)	0.69
AST(IU/L)	33.0(25.0-65.0)	47.0(38.2-73.7)	0.07
ALT(IU/L)	17.0(12.0-34.0)	26.0(10.7-42.7)	0.30
Lactic acid (mmol/L)	2.7(1.3-4.7)	3.8(3.0-7.4)	0.001
BNP (pg/ml)	210.0(96.2-480.5)	427(192.7-902.0)	0.07
CRP(mg/dl)	101.3(39.9-216.6)	80.7(52.6-151.1)	0.43
Procalcitonin(ng/ml)	0.5(0.1-5.9)	0.92(0.16-7.9)	0.57

Data are presented as the median(interqurtile range(IQR)) for continuous variables and as the number(%) for the categorical variables.

SOFA: Sequential Organ Failure Assessment, APACHE: Acute Physiology and Chronic Health Evaluation MV: Mechanical Ventilation, CRRT: Continuous renal replacement therapy, BP: Blood pressure HR: Heart rate RR: Respiratory rate BT: Body temperature, WBC: White blood cell, DNI 1: Delta neutrophil index at 24 hour of ICU admission, DNI 2: Delta neutrophil index at 48 hour of ICU admission, DNI 3: Delta neutrophil index at 72 hour of ICU admission, Hb: Hemoglobin, Hct: Hematocrit, Na:Sodium, K: Potassium, BUN:Blood urea nitrogen, Cr: Creatinine, AST: Aspartate aminotransferase, ALT:Alanine aminotransferase, Lactic acid 4: Lactic acid at 4hr of ICU admission, BNP: Brain natriuretic peptide, CRP: C- reactive protein

Table S3. Cox proportional hazard analysis of predictive factor for 28-day mortality

Variables	Exp(B)	95% CI	p-value
DNI 3(%)	1.072	1.010-1.150	0.048
Sepsis (via septic shock)	5.210	1.318-20.592	0.020

DNI 3: Delta neutrophil index at 72hours of ICU admission
