# Supplementary figures and images for: A Usefulness of Delta Neutrophil Index (DNI) for Prediction of 28 Day Mortality in Patients with Pneumonia-Induced Sepsis in the Intensive Care Unit
Source: J Clin Med. 2025 Mar 15;14(6):2002. doi: 10.3390/jcm14062002 (PMC11942979; doi:10.3390/jcm14062002)

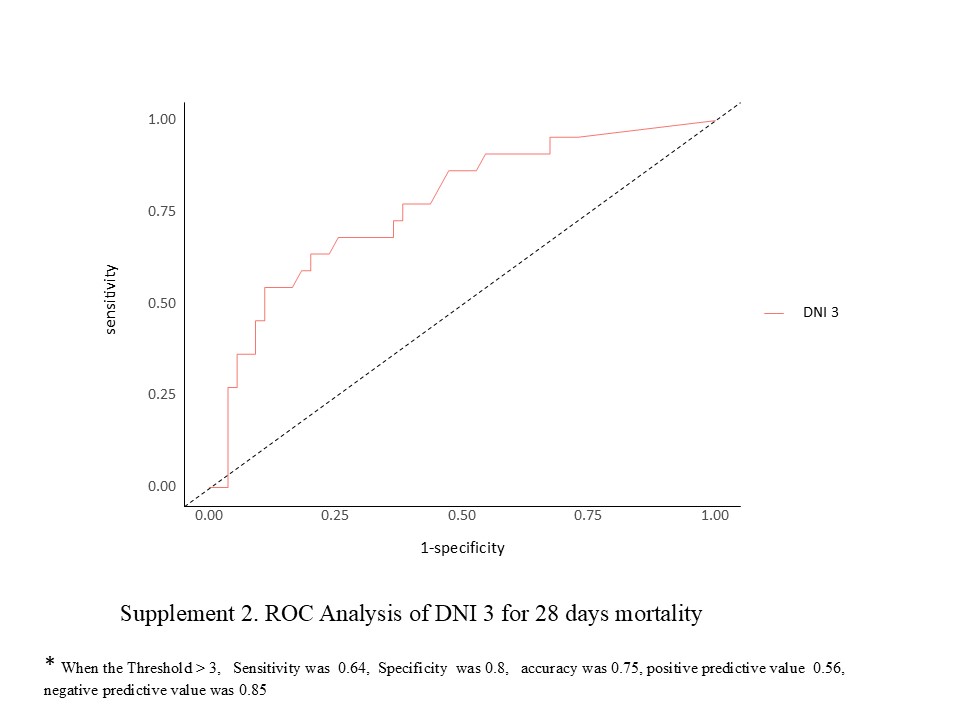

Supplement: Supplementary file 1 [file jcm-14-02002-s001.zip › supplement 2.jpg]
